# Resting spontaneous baroreflex sensitivity and cardiac autonomic control in anabolic androgenic steroid users

**DOI:** 10.6061/clinics/2018/e226

**Published:** 2018-05-15

**Authors:** Marcelo R. dos Santos, Ana L.C. Sayegh, Rafael Armani, Valéria Costa-Hong, Francis R. de Souza, Edgar Toschi-Dias, Luiz A. Bortolotto, Mauricio Yonamine, Carlos E. Negrão, Maria-Janieire N.N. Alves

**Affiliations:** I Instituto do Coracao (InCor), Hospital das Clinicas HCFMUSP, Faculdade de Medicina, Universidade de Sao Paulo, Sao Paulo, SP, BR; II Toxicologia, Faculdade de Ciencias Farmaceuticas, Sao Paulo, SP, BR; III Faculdade de Educacao Fisica e Esporte, Universidade de Sao Paulo, Sao Paulo, SP, BR

**Keywords:** Autonomic Nervous System, Blood Pressure, Baroreflex Sensitivity, Pulse Wave Velocity

## Abstract

**OBJECTIVES:**

Misuse of anabolic androgenic steroids in athletes is a strategy used to enhance strength and skeletal muscle hypertrophy. However, its abuse leads to an imbalance in muscle sympathetic nerve activity, increased vascular resistance, and increased blood pressure. However, the mechanisms underlying these alterations are still unknown. Therefore, we tested whether anabolic androgenic steroids could impair resting baroreflex sensitivity and cardiac sympathovagal control. In addition, we evaluate pulse wave velocity to ascertain the arterial stiffness of large vessels.

**METHODS:**

Fourteen male anabolic androgenic steroid users and 12 nonusers were studied. Heart rate, blood pressure, and respiratory rate were recorded. Baroreflex sensitivity was estimated by the sequence method, and cardiac autonomic control by analysis of the R-R interval. Pulse wave velocity was measured using a noninvasive automatic device.

**RESULTS:**

Mean spontaneous baroreflex sensitivity, baroreflex sensitivity to activation of the baroreceptors, and baroreflex sensitivity to deactivation of the baroreceptors were significantly lower in users than in nonusers. In the spectral analysis of heart rate variability, high frequency activity was lower, while low frequency activity was higher in users than in nonusers. Moreover, the sympathovagal balance was higher in users. Users showed higher pulse wave velocity than nonusers showing arterial stiffness of large vessels. Single linear regression analysis showed significant correlations between mean blood pressure and baroreflex sensitivity and pulse wave velocity.

**CONCLUSIONS:**

Our results provide evidence for lower baroreflex sensitivity and sympathovagal imbalance in anabolic androgenic steroid users. Moreover, anabolic androgenic steroid users showed arterial stiffness. Together, these alterations might be the mechanisms triggering the increased blood pressure in this population.

## INTRODUCTION

Misuse of anabolic androgenic steroids (AASs) in bodybuilding athletes is a common strategy to enhance strength and skeletal muscle hypertrophy. However, AASs are associated with negative impacts on health, such as muscle sympathetic hyperactivation, reduced vasodilatation, and increased blood pressure (BP) 1-3. On the other hand, the exact mechanisms involved in these alterations in AAS user (AASU) athletes are still unknown.

In several cardiovascular diseases such as hypertension and heart failure, lower baroreflex sensitivity (BRS) is one of the main mechanisms leading to increased sympathetic hyperactivation 4-6. Increased systemic arterial pressure activates the arterial baroreceptors leading to bradycardia and decreased peripheral vascular resistance (parasympathetic predominance). On the other hand, decreased systemic arterial pressure deactivates the baroreceptors leading to tachycardia and an increase in vascular resistance (sympathetic predominance) 5.

In a previous microneurography study, it was demonstrated that AASUs showed higher muscle sympathetic nerve activity (MSNA) than AAS nonusers (AASNUs) 2. In fact, it was demonstrated by single linear regression analysis that the mean BP had a strong correlation with MSNA. Moreover, a decreased resting forearm blood flow was found in AASUs 2. Experimental studies support the concept that rats treated with AASs showed hypertension associated with impaired BRS control of the heart rate 7. Consequently, the increased BP observed in AASU athletes could be related to impaired BRS, which is used to control BP.

Likewise, rats treated with high doses of AASs showed dysfunction in tonic cardiac autonomic regulation, as evaluated by spectral analysis of heart rate variability 8,9. These results demonstrated the existence of a relationship between AAS misuse and imbalance in autonomic control (of both the periphery and the heart).

Therefore, the present study was performed to test whether AASU athletes showed impaired BRS and an imbalance in cardiac sympathovagal control. In addition, we evaluated pulse wave velocity (PWV) to ascertain the arterial stiffness of large vessels.

## MATERIALS AND METHODS

### Study design

This is a prospective, cross-sectional comparison study of AASUs and AASNUs. This study was designed to ascertain the influence of AASs on BRS, cardiac autonomic control, and PWV. To show the effect of AASs on these parameters, all the measurements were obtained when the subjects were at the peak of their AAS use. To avoid the influence of exercise on the measurements, all participants were instructed to refrain from exercise 48 hours before the experimental protocol. Moreover, they abstained from caffeine-containing products, fatty foods, and any sports supplements for at least 24 hours before the measurements.

In the present study, from November 2013 to January 2015, 14 male AASUs and 12 healthy age-matched male AASNUs were invited to participate.

Anamnesis was taken from all participants to obtain information about their exercise training regime, AAS usage, medications, and eating habits, e.g., use of dietary supplements and percentages of macronutrients (e.g., proteins and carbohydrates).

The inclusion criteria for both groups were 1) healthy subjects and 2) strength training (hypertrophy) for at least 2 years. Exclusion criteria for both groups were 1) smoking, 2) alcohol intake, 3) use of medication (e.g., diuretics and anti-hypertensives), and 4) liver and kidney disease.

All the measurements were obtained when the subjects were at the peak of their AAS use (between 4-6 weeks, self-administering, maximum dosage, and self-reported). The AAS cycles of the AASU group lasted from 8-12 weeks. In addition, the subjects had been using AASs for at least 2 years with 2-4 cycles per year. The self-reported most used AASs were stanozolol, testosterone propionate, testosterone cypionate, nandrolone decanoate, boldenone undecylenate, and methenolone enanthate.

The study protocol was approved by the Local Human Subject Protection Committee (number 569.389), and written consent was given by each individual. The study was performed in accordance with the principles of the Declaration of Helsinki.

All participants were enrolled in traditional bodybuilding strength-training exercises consisting of 3-4 sets of 8-12 repetitions for approximately 2-4 exercise muscle groups 5 days per week. Division of the training consisted of 2-3 muscle groups exercised per day (e.g., training A: chest, triceps brachii, and shoulders; training B: back and biceps brachii; training C: legs). All participants were asked about their perceived exertion during the training sessions, which should have been 8 to 10 (hard to extremely hard) in accordance with OMNI-Resistance Exercise Scale (OMNI-RES) 10. Both groups had trained for 8 years on average.

### Heart rate, arterial pressure, and respiratory rate

For 10 minutes of baseline recording, the heart rate was monitored continuously through lead II of an electrocardiogram. Simultaneously, arterial blood pressure (systolic blood pressure, SBP; diastolic blood pressure, DBP; and mean blood pressure, MBP) was monitored using a Finometer PRO^®^ (Finapress Medical System), which provides noninvasive measurement of the beat-to-beat BP. Respiratory rate was monitored using a piezoelectric thoracic belt (Pneumotrace II, model 1132, Respiration Transducer, UFI, USA) placed around the upper abdomen.

Spontaneous BRS was determined using the sequence method 11, which consists of the identification of 3 or more consecutive beats in which progressive increases or decreases in SBP are followed by progressive lengthening or shortening of the RR interval during spontaneous behavior. Consecutive and concomitant BP increases represent spontaneous activation of the baroreceptors (up sequences; BRS+), and decreases in BP represent spontaneous deactivation of the baroreceptors (down sequences; BRS-). The threshold values for including beat-to-beat SBP and RR interval changes in a sequence were set at 1 mm Hg and 3 ms, respectively. The reflex sensitivity was evaluated by computing the slope of the regression line between changes in SBP and RR interval. The average of all computed slopes was used to obtain the BRS 5. Finometer PRO^®^ has a 1 second delay to convert the analogic signal to digital signal. To correct this delay between RR interval and SBP, we extracted similar and correspondent amounts of RR interval and SBP. Signals refer to sequences during which the SBP and RR interval of the following beat changed in the same direction, either increasing or decreasing (baroreflex sequences).

### Cardiac autonomic evaluation

The electrocardiogram signal to extract the time series for the heart rate (RR interval) and the beat-to-beat BP were recorded with a computer program (Windaq, sampling frequency of 500 Hz and a resolution of 16 bits). The cardiovascular RR interval fluctuation was assessed in the frequency domain using autoregressive spectral analysis as described previously 12,13. For stationary segments of the time series, autoregressive parameters were assessed via Levinson–Durbin recursion, and the order of the model was selected according to Akaike’s criterion 12,13. After that, autoregressive spectral decomposition was performed. This procedure permitted the automatic quantification of the center frequency and the power of each relevant component in absolute normalized units (n.u.). Low frequency (LF) components (0.04-0.15 Hz) indicated the predominance of sympathetic modulation. High frequency (HF) components (0.15 and 0.4 Hz), synchronized with the breathing signals, were considered parasympathetic modulation. The power spectrum (absolute; abs.) is indicated in ms^2^, and the power spectral density is calculated in n.u. The normalization was performed by dividing the power of the LF or HF component by the total spectral power, as previously described 12,13. The ratio of LF to HF (LF/HF) was calculated as cardiac sympathovagal balance.

The breathing was spontaneous, and the breathing of 2 AASU and 1 AASNU subjects was uncoupled in the HF range; these subjects were excluded from the cardiac autonomic evaluation.

### Pulse wave velocity (PWV)

Carotid-femoral PWV was measured using a noninvasive automatic device (Complior SP®, Artech Medical, Pantin, France) as previously described 14. The pressure waveforms were recorded in the common carotid artery and femoral artery using a pressure-sensitive transducer. The PWV was calculated by the formula: PWV=D/t, where “D” means the distance between the recording sites, and “t” is the pulse transit time. During 10 different cardiac cycles, the measurements were repeated, and the mean was used in the analysis. The experiment was performed as previously suggested 15.

### Experimental protocol

All procedures were performed in a quiet, temperature-controlled (21°C) room in the morning at approximately the same time of day. All measurements were assessed in the supine position. The participants were instructed to restrain from exercise 48 hours before the experimental protocol and to eat their breakfast normally. However, they abstained from caffeine-containing products, fatty foods, and any sports supplements for at least 24 hours before the measurements.

### Statistics

Normality (Shapiro-Wilk) and homogeneity (Levene) tests were conducted to test the distribution of the data. Data are presented as the mean ± SD or median (interquartile range – IQR – 25%-75%). Possible differences between groups were analyzed using an unpaired Student’s t-test or Mann-Whitney-Wilcoxon test for parametric or nonparametric data, respectively.

Univariate correlation (Pearson correlation) between MBP, age, BRS-, BRS+, mean BRS, PWV, LF, HF, and LF/HF was conducted to test the association between parameters.

An analysis of covariance (ANCOVA) was conducted to test the possible influence of age and body mass index (BMI) on MBP and mean BRS. Differences were considered significant at *p*<0.05. All data were analyzed using SPSS version 18.0.

## RESULTS

The physical characteristics, heart rate, BP, and PWV are shown in Table 1. There were no significant differences between AASUs and AASNUs in age and height. However, AASUs showed higher weight, BMI, heart rate, SBP, DBP, MBP, and PWV than AASNUs. After controlling for cofactors, ANCOVA showed no significant differences in MBP for age (*p*=0.47) or BMI (*p*=0.13).

The spontaneous BRS and heart rate variability are shown in Table 2. The mean spontaneous BRS, spontaneous BRS+, and spontaneous BRS- were lower in AASUs than in AASNUs. After controlling for cofactors, ANCOVA showed no significant differences in mean spontaneous BRS for age (*p*=0.23) or BMI (*p*=0.94). For the heart rate variability, there were no significant differences between AASUs and AASNUs in the variance and LF_abs_. Notably, AASUs showed lower HF_abs_ and HF_n.u._, whereas LF_n.u._ was higher, indicating cardiac sympathetic hyperactivation and loss of cardiac parasympathetic control in this group.

Single linear regression analysis showed significant correlations between MBP and BRS+, mean BRS, and PWV (Table 3). However, age, BRS-, LF, HF, and LF/HF were not correlated with MBP (Table 3).

## DISCUSSION

Our study evaluated the influence of AASs on BRS and cardiac autonomic control to investigate their potential roles in the mechanisms underlying increased BP in athletes misusing AASs. In addition, we evaluated arterial stiffness by PWV. Our main findings were that spontaneous BRS and cardiac sympathovagal balance were impaired in AASUs compared to AASNUs. Moreover, we found a correlation between BP and spontaneous BRS and arterial stiffness.

In older subjects, a reduction in arterial compliance is one of the main mechanisms of impaired cardiovagal BRS 16,17. Baroreceptors are located at the carotid sinus and aortic arch, and these receptors can be influenced by the compliance of these elastic arteries 18, which could explain, at least in part, the decreased BRS observed in our study.

Baroreceptors control moment-to-moment variations in BP by mechanical transduction of pressure into barosensory vessel stretch and neural transduction of stretch into vagal outflow 19,20. BRS inefficiency in hypertension leads to sympathetic hyperactivity and increased BP 21. In the present study, although the BP of AASUs was not in the hypertension range (SBP/DBP: 127/75 mmHg), it was different from that of the age-matched control group (SBP/DBP: 117/66 mmHg). The beat-to-beat BP measurement in this study was performed in a quiet room with the subjects lying down for the baseline condition, which does not reflect their daily life. A higher 24-hour BP has been observed in AASUs 2, and this result may have greater relevance to real life. Somehow, this slight increase in BP could be associated with alterations in BRS in this population.

Another interesting finding of our study was that AASUs showed higher PWV than AASNUs. Assessment of arterial stiffness is a recommended measurement in the European Society of Cardiology guidelines because PWV is associated with future fatal and nonfatal cardiovascular events, even in younger people 22. In our study, this parameter was different in AASUs, and it may indicate increased risk for cardiovascular disease. Arterial stiffness is directly correlated with poor cardiac outcomes in the general population 22. PWV provides information about arterial segment stiffness 23, and in the present study, PWV was higher, suggesting that arterial stiffness worsens hemodynamic problems in young male AASUs.

Autonomic imbalance in AASUs, especially muscle sympathetic hyperactivation, was previously demonstrated 2; however, whether AASs could alter cardiac autonomic control in humans was unknown. As demonstrated in the present study, the cardiac sympathetic and parasympathetic activities were impaired in AASUs. Indeed, we observed cardiac sympathetic predominance, as indicated by the elevated LF n.u. and LF/HF, in AASUs than in AASNUs. Elevated sympathetic nerve activity is known to increase cardiovascular risk and mortality in some pathological conditions, such as hypertension 24 and heart failure 25. Previous studies showed higher BP at rest 2 and during exercise 26 in AASUs, and elevated MSNA was positively correlated (r=0.75, *p*=0.002) with elevated mean 24-hour BP 2, suggesting a potential mechanism for hemodynamic alteration. Moreover, cardiac parasympathetic control, as measured by the HF component (n.u.), was significantly lower in the AASUs than in AASNUs. This result is consistent with a recent study that showed that AASUs had lower heart rate recovery after maximal exercise 27, which is a marker of poor vagal reactivation 28. Taken together, these results (delay in heart rate recovery and lower HF) show impaired cardiac parasympathetic control in AASUs.

There are several studies showing that higher BMI in overweight and obese populations is associated with increased sympathetic nerve activity 29. In our study, we found that AASUs presented with BMIs of 29±3 kg/m^2^, which could influence sympathetic outflow and, consequently, BP. However, when we performed an ANCOVA to test the possible influence of BMI on MBP, we observed no significant differences (*p*=0.13), showing that BMI is not correlated with higher BP in AASUs. In fact, higher BMI in bodybuilders is due to higher muscle mass than fat mass 30.

We recognized limitations in our study. We do not know if these results persist if the baroreflex is tested using vasoactive drugs, such as phenylephrine and sodium nitroprusside. We did not compare our results to a sedentary control group, and we are cautious about drawing conclusions from our study group only compared to a strength-training exercise group without AAS use. However, our results show alterations in BRS, cardiac autonomic nervous system, and BP in AASUs. Someone could argue that strength-training exercise, especially acute bouts of exercise, could influence arterial stiffness. However, all participants were instructed to refrain from exercise 48 hours before the experimental protocol. We did not perform body composition measurements in the participants. However, all of them were strength athletes, and they visually presented large muscle mass and low fat composition. Finally, although we demonstrated a correlation between BP and BRS in AASUs, we cannot at this moment support a cause-effect relationship. Future studies should be designed to address this issue.

In conclusion, our results provide evidence for lower BRS and sympathovagal imbalance in AASUs. Moreover, AASUs showed arterial stiffness. Together, these alterations might be the mechanisms triggering the increased BP in this population.

## AUTHOR CONTRIBUTIONS

Santos MR, Sayegh AL, Armani R and Alves MJ were responsible for the study design, manuscript writing and statistical analysis. Costa-Hong V, Souza FR, Toschi-Dias E, Bortolotto LA, Yonamine M and Negrão CE were responsible for the writing of the Methods section and manuscript review.

## Figures and Tables

**Table 1:** Physical characteristics, heart rate, blood pressure, and pulse wave velocity in anabolic androgenic steroid users and anabolic androgenic steroid nonusers.

	AASU	AASNU	*p*
N	14	12	
Age, years	34±7	30±4	0.11
Weight, kg	89±8	80±5	0.002
Height, m	1.76±0.07	1.78±0.07	0.50
BMI, kg/m^2^	29±3	25±2	0.001
Heart rate, bpm	68±9	56±7	0.002
SBP, mmHg	127±13	117±7	0.03
DBP, mmHg	75±7	66±5	0.003
*MBP, mmHg	94±9	84±6	0.005
PWV, m/s	9.56±0.91	8.58±1.20	0.03

* Values ANCOVA adjusted (for age and BMI). Data are presented as the mean±SD. AASNU=anabolic androgenic steroid nonuser, AASU=anabolic androgenic steroid user, BMI=body mass index, SBP=systolic blood pressure, DBP=diastolic blood pressure, MBP=mean blood pressure, PWV=pulse wave velocity.

**Table 2:** Spontaneous baroreflex sensitivity and cardiac autonomic control in anabolic androgenic steroid users and anabolic androgenic steroid nonusers.

	AASU	AASNU	*p*
N	14	12	
Baroreflex sensitivity			
*Mean BRS, ms/mmHg	15.31 (8.36-16.73)	22.87 (14.57-25.63)	0.01
BRS+, ms/mmHg	14.68 (8.61-17.47)	24.07 (11.56-31.43)	0.03
BRS-, ms/mmHg	13.47±3.91	19.25±5.68	0.005
N	12	11	
Cardiac autonomic control RRi			
Variance, ms^2^	3.264 (1604-4820)	3.463 (1717-6056)	0.53
LF_abs._, ms^2^	715 (370-1358)	1.269 (949-1406)	0.16
HF_abs._, ms^2^	377 (138-651)	746 (432-2313)	0.03
HF, n.u., %	36.37 (25.91-40.90)	40.44 (28.60-63.11)	0.04
LF, n.u., %	67.10 (59.75-76.67)	59.55 (36.88-71.40)	0.04
LF/HF	2.40 (1.44-3.45)	1.40 (0.55-2.55)	0.04

* Values ANCOVA adjusted (for age and BMI). Data are presented as the mean±SD or median±IQR (25%-75%; IQR=interquartile range). AASNU=anabolic androgenic steroid nonuser, AASU=anabolic androgenic steroid user, BRS+=spontaneous baroreflex sensitivity increases, BRS-=spontaneous baroreflex sensitivity decreases, RRi=R-R interval, LF=low frequency, HF=high frequency, abs.=absolute, n.u.=normalized unit.

**Table 3:** Single linear regression (Pearson) with mean blood pressure serving as the dependent variable.

Variables	R	P
Age, years	0.28	0.11
BRS+, ms/mmHg	-0.41	0.03
BRS-, ms/mmHg	-0.23	0.15
Mean BRS, ms/mmHg	-0.38	0.04
PWV, m/s	0.49	0.01
LF, n.u., %	0.28	0.11
HF, n.u. %	-0.28	0.11
LF/HF	0.33	0.64

BRS+=spontaneous baroreflex sensitivity increases, BRS-=spontaneous baroreflex sensitivity decreases, PWV=pulse wave velocity, LF=low frequency, HF=high frequency, n.u.=normalized unit.
